# Analysis of Beijing Douzhir Microbiota by High-Throughput Sequencing and Isolation of Acidogenic, Starch-Flocculating Strains

**DOI:** 10.3389/fmicb.2018.01142

**Published:** 2018-05-29

**Authors:** Yunhe Xu, Yang Yu, Yumin Tian, Yuhong Su, Xiaona Li, Zhen Zhang, Hongyan Zhu, Jie Han, Huajiang Zhang, Liying Liu, Lili Zhang

**Affiliations:** ^1^Department of Food Science and Engineering, Jinzhou Medical University, Jinzhou, China; ^2^College of Food Science, Shenyang Agricultural University, Shenyang, China; ^3^School of Food Science, Northeast Agricultural University, Harbin, China

**Keywords:** Douzhir, Ma tofu, high-throughput sequencing, isolation, starch flocculation, acidogenic

## Abstract

Beijing Douzhir is a traditional Chinese fermented drink produced by the natural fermentation of mung beans as the raw material. Ma tofu is an edible by-product of Douzhir processing. Douzhir microbiota, particularly bacteria involved in the natural fermentation process, has not been clearly established, resulting in limited industrial Douzhir production. Here, three uncooked Douzhir samples (D group) and three uncooked Ma tofu samples (M group) (two replicates per sample) were collected from three manufacturers in different locations in Beijing. The composition and diversity of the bacterial communities in each sample were analyzed by high-throughput sequencing. In total, 637 operational taxonomic units (OTUs) were revealed in the D group through database alignment, and 656 OTUs were found in the M group. The Chao, ACE, and Shannon indices were not significantly different in Douzhir samples from different manufacturers (*p* > 0.05). Representatives of six phyla were found in all 12 samples. Dominant bacteria were isolated and identified using mung bean juice as the growth medium. In both Douzhir and Ma tofu samples, dominant bacteria belonging to Firmicutes and Proteobacteria comprised > 94% of the total microbiota. The dominant bacteria included members of the *Lactococcus, Acetobacter, Streptococcus*, and *Lactobacillus* genera. Considering the dominant-microbiota information, we employed a plate-separation technique and isolated two strains of acid-producing bacteria from the Douzhir and Ma tofu samples with starch-flocculating activity: *Acetobacter indonesiensis* and *Lactococcus lactis* subsp. *lactis*. Such strains can serve as a foundation for the standardized industrial production of Douzhir.

## Introduction

**FIGURE 1:**
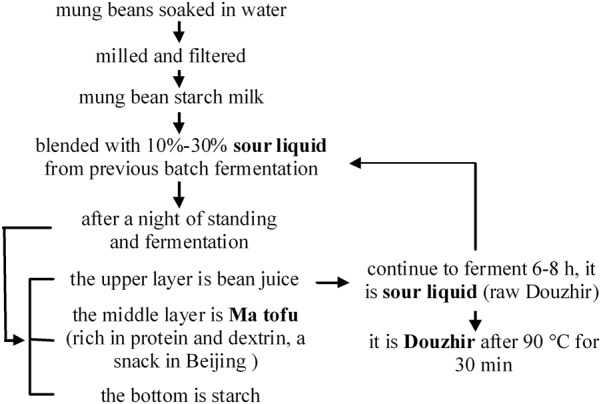
Production of mung bean starch by sour liquid flocculation.

Douzhir (fermented mung bean juice) has been consumed for at least 1000 years and is currently the most popular fermented snack in Beijing. Douzhir comprises residual material left after starch production from mung beans. This sour liquid is rich in proteins, vitamin C, and dietary fiber (Ding et al., 2010). Douzhir possesses appetizing, heat-relieving, detoxifying, and other beneficial properties that account for its popularity (Chen et al., 2013; Deng et al., 2013; Miao et al., 2013; Wu et al., 2015). **Figure 1** shows the technology used for mung bean starch processing. It has been reported that microorganisms present in the sour liquid are responsible for starch flocculation (Zhang et al., 2017). Adding sour liquid is a key step in Douzhir processing that serves two purposes. Firstly, this treatment accelerates starch settling: when sour liquid is added to mung bean starch milk, starch rapidly coagulates into floc aggregates and rapidly settles. Secondly, sour liquid is added to the mixture to ferment the bean juice and make it taste sourer. Flocculated starch possesses a relatively high specific density and therefore occupies the lowest position in the container. The “Ma tofu” fraction contains protein and dextrin, and settles above the starch fraction. The top layer comprises gray-green raw bean juice (Liu and Shen, 2007a,b).

Douzhir is produced through spontaneous fermentation, which is greatly influenced by environmental factors, such as the composition of the raw material, temperature, and pH. Excessive or insufficient fermentation due to the instability of such factors leads to inconsistent product quality. If fermentation broth lacks starch-flocculating activity, then the starch will not separate completely, which seriously affects the Douzhir quality (Cocolin et al., 2000; Li et al., 2008). Starch flocculation and acid production result from the activity of microorganisms in the sour liquid (Chang et al., 2006; Ding et al., 2009; Hamady and Knight, 2009; Escobar-Zepeda et al., 2016). Therefore, the industrialisation and standardization of Douzhir production should take into account the composition of the microbiota in the sour liquid. In addition, screening for the presence of acid-forming bacteria critical for starch flocculation may be useful. In this study, the V4 regions of the 16S rRNA genes of bacteria present in Douzhir and Ma tofu were analyzed by high-throughput sequencing, in conjunction with traditional microbial-isolation and culturing techniques, to determine the dominant microorganisms with starch-binding and flocculating activities. We isolated Douzhir fermentative bacteria that accelerated starch deposition and acid yield. Such strains can serve as a foundation for standardizing the industrial production of Douzhir.

## Materials and Methods

### Sample Collection

Three samples of uncooked Douzhir and three samples of uncooked Ma tofu (two replicates per sample) were collected from three manufacturers in different locations in Beijing (DZ, LFS, LCQK). Douzhir samples were named with the letter “D” (D-DZ-1, D-DZ-2; D-LFS-1, D-LFS-2; D-LCQK-1, D-LCQK-2) (D group), whereas Ma tofu samples were named with the letter “M” (M-DZ-1, M-DZ-2; M-LFS-1, M-LFS-2; M-LCQK-1, M-LCQK-2) (M group). The 12 samples were preserved in liquid nitrogen and subsequently used for DNA extraction and polymerase chain reaction (PCR) amplification. Isolation of bacteria was performed immediately after sampling.

### Bacterial 16S rDNA Sequencing

Microbial genomic DNA was extracted from 12 replicates by using a TIANamp DNA Stool Kit (TIANGEN Biotech, catalog #DP328) according to the manufacturer’s instructions^1^. The hyper-variable V4 region of the 16S rDNA gene was amplified using the following universal primers: 520F (5′-AYTGGGYDTAAAGNG-3′) and 802R (5′-TACNVGGGTATCTAATCC-3′) (Claesson et al., 2009). PCR was performed with an initial 30-s denaturation at 98°C; 25 cycles of denaturation at 98°C for 30 s, annealing at 50°C for 30 s, and extension at 72°C for 30 s; and a final extension step at 72°C for 5 min. The PCR product was purified using a Quick Gel Extraction Kit (Qiagen, catalog #28706). The PCR product from each sample was used to construct a sequencing library using the Illumina TruSeq DNA Sample Preparation Kit and the TruSeq Library Construction Kit. For each sample, barcoded V4 PCR amplicons were sequenced using the Illumina MiSeq platform.

Sequence reads were discarded under the following circumstances: sequence length less than 150 base pairs (bp), average Phred score less than 20, presence of ambiguous base contents, more than six homopolymer runs, or the presence of mismatched primers. Afterward, the obtained sequences were passed through a quality filter and assembled using Flash software^2^, which required overlapping of read 1 and read 2 by ≥10 bp and the absence of mismatches. Unassembled reads were discarded. Chimera sequences were removed using UCHIME in MOTHUR (version 1.31.2)^3^. Amplification and sequencing of the 16S rDNA V4 hyper-variable region were completed by Personal Biotechnology Co., Ltd. (Shanghai, China).

### Operational Taxonomic Unit (OTU) Clustering

Sequence clustering was performed using the UCLUST algorithm in QIIME^4^, and the results were clustered into OTUs. The longest sequence in each cluster was selected as the representative sequence. The taxonomy of each OTU was assigned by BLAST searching the representative sequence against Greengenes reference database (Release 13.8)^5^. Unknown archaea or eukaryotic sequences were filtered and removed. ACE, Chao, and Shannon indices were calculated using the summary single command in MOTHUR. A Venn diagram of the between-group OTUs was generated using the limma package of R software. Non-metric multidimensional scaling (NMDS) plots of sequence-read abundances were generated with the Vegan package in R.

### Isolation of Bacterial Strains

Bacterial strains were isolated by serial dilution and plating techniques. Mung bean juice medium containing 1% calcium carbonate was prepared as follows. Twenty grams of mung beans was boiled in 1 L of distilled water for 20 min, and the resulting broth was then decanted or strained. Distilled water was then added to the suspension to obtain a total volume of 1 L. Then, 20 g of glucose, 2 g of lactose, 5 g of sodium acetate, 5 g of yeast extract, and 2 g of K_2_HPO_4_ were added, and the medium was sterilized by autoclaving at 1.05 kg/cm^2^ for 15 min. Twenty-five milliliters of each sample was suspended in 225 mL of sterile normal saline, homogenized in an incubator with shaking at 30°C and 150 rpm for 5 min, and serially diluted from 10^-1^ to 10^-8^. Next, 0.1 mL of each dilution was spread onto mung bean juice medium-agar plates containing 1% calcium carbonate and incubated at 30°C for 24–48 h (Ryan et al., 2006). After incubation, presumptive bacterial colonies (colonies with calcium carbonate-dissolution rings) were obtained from suitably diluted plates and transferred onto freshly prepared plates until pure cultures were obtained.

### Flocculation-Rate (FR) Measurements

Isolated strains were added to mung bean juice medium for 24 h and cultured at 30°C. After culturing, the pH of the fermented liquid was measured and the FR was determined.

To measure the FR, 100 mL of distilled water, 0.4 g of mung bean starch, and 2 mL of fermented liquid were placed in a 150-mL beaker. The liquid was agitated for 1 min on a magnetic stirrer and then left to stand for 3 min. As a control, distilled water was used instead of fermented liquid. The flocculation efficiency was expressed as the FR by measuring the decrease of turbidity of the upper phase, using the following equation:

$$\begin{array}{c} FR(\%)=\frac{A-B}{A}\times 100\% \end{array}$$

where A and B are optical densities of the control and sample, respectively, at 550 nm.

### Amplification and Sequencing of 16S rDNA

Isolates were grown until late stationary phase in 5 mL of medium. Cultures were centrifuged for 10 min at 4,000 × *g*. Each cell pellet was resuspended in 0.5 mL of distilled water, and DNA was extracted using a TIANamp DNA Stool Kit, according to the manufacturer’s instructions.

Full-length 16S rDNA amplicons were generated using primers 27F (5′-AGAGTTTGATCCTGGCTCAG-3′ and 1492R (5′-CTACGGCTACCTTGTTACGA-3′). The PCR thermocycling conditions were as follows: an initial denaturation at 95°C for 5 min, 35 cycles of (95°C for 30 s, 58°C for 30 s, and 72°C for 90 s), and a final extension step of 72°C for 7 min. PCR products were purified and sequenced using an ABI 3730 automated sequencer at Personal Biotechnology Co., Ltd. (Shanghai, China).

All sequences were matched against most similar 16S rDNA nucleotide sequences in GenBank using the BLASTN program^6^ (Altschul et al., 1997). Bacterial identification was assumed when the query sequence showed > 97% similarity with the target 16S rRNA gene sequence (Gevers et al., 2005; Guo et al., 2010). The 16S rDNA sequences were aligned using CLUSTAL W, and a phylogenetic tree was constructed using the neighbor-joining method (MEGA 5.0). Bootstrap resampling was carried out with 1,000 replications to estimate the confidence of the tree topologies.

### Microscopic Observations of Starch Granules With Attached Bacteria

The distribution of mung bean starch particles was observed by optical microscopy before and after the addition of fermentation liquid from selected strains. Samples of starch granules with attached bacterial cells were fixed with a 3% glutaraldehyde solution (v/v) in 0.01 M phosphate buffer (pH 7.2) onto brass stubs. Next, they were chromium-coated (Xenosput 2000 chromium coater) using deposition parameters of 0.06 sputter amps for 40 s. Coated preparations were visualized with a Hitachi S900 scanning electron microscope at an accelerating voltage of 2 kV. A minimum of eight granules were visualized per field. All cells visibly attached to the granules were counted (O’Riordan et al., 2001).

### Statistical Analysis

The diversity index, FR, and pH values are expressed as the mean ± standard error of the mean. Statistical analyses were performed to determine significant differences (*p* < 0.05). Differences between groups were assessed using one-way analysis of variance followed by the least significant difference test. All statistical analyses were performed using SPSS software, version 16.0 (Xu et al., 2016).

## Results

### OTU Clustering and Annotation

**FIGURE 2:**
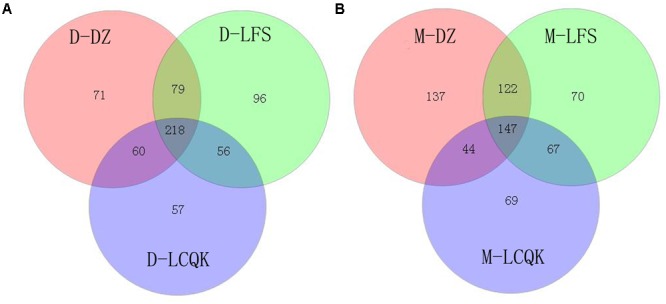
Analysis of operational taxonomic units (OTUs) shared by different groups. **(A)** We identified 428, 449, and 391 OTUs in the D-DZ, D-LFS, and D-LCQK groups, respectively. The data showed that 297 OTUs were shared between the D-DZ and D-LFS groups; 278 were shared between the D-DZ and D-LCQK groups, and 274 were shared between the D-LFS and D-LCQK groups. The total shared richness was 218 OTUs, and the total richness of all was 637 OTUs. **(B)** We identified 450, 406, and 327 OTUs in the M-DZ, M-LFS, and M-LCQK groups, respectively. The data showed that 269 OTUs were shared between the M-DZ and M-LFS groups, 191 were shared between the M-DZ and M-LCQK, and 214 were shared between the M-LFS and M-LCQK groups. The total shared richness was 147 OTUs. The total richness of all groups was 656 OTUs.

Trimmed and assembled sequences were clustered at 97% similarity using UCLUST from QIIME. We identified 637 OTUs in the D group through database alignment by BLAST analysis in QIIME. The distribution of OTUs in the D group was as follows: 428 were found in the D-DZ sample, 449 were found in the D-LFS sample, and 391 were found in the D-LCQK sample (**Figure 2A**). In addition, 656 OTUs were found in the M group: 450 in the M-DZ sample, 406 in the M-LFS sample, and 327 in the M-LCQK sample (**Figure 2B**). The D and M groups had total shared-richness values of 218 and 147, respectively (**Figure 2**). Douzhir and Ma tofu samples exhibited similar numbers of OTUs, but samples from different manufacturers varied with respect to the number of unique OTUs. The Chao, ACE, and Shannon indices did not significantly vary among the Douzhir samples (*P* > 0.05) (**Table 1**), indicating minimal differences in richness and evenness for the microorganisms in the liquids obtained from different manufacturers. The Shannon index of the M-LCQK sample was significantly lower (*P* < 0.05) than those of the M-DZ and M-LFS samples (**Table 2**). The evenness values of the Ma tofu microorganisms were significantly different in samples from different manufacturers (*P* < 0.05).

**Table 1:** Douzhir microbiota diversity indices.

Sample	Chao	ACE	Shannon
D-DZ	453.22 ± 27.87^a^	492.31 ± 24.39^a^	1.98 ± 0.0015^a^
D-LFS	466.97 ± 31.65^a^	539.23 ± 87.42^a^	2.11 ± 0.2004^a^
D-LCQK	432.71 ± 10.25^a^	520.35 ± 100.86^a^	2.31 ± 0.0241^a^

*Means within a column followed by different letters are significantly different (*p* < 0.05).*

**Table 2:** Ma tofu microbiota diversity indices.

Sample	Chao	ACE	Shannon
M-DZ	481.47 ± 35.32^a^	583.85 ± 52.09^a^	2.16 ± 0.10^b^
M-LFS	436.14 ± 28.74^a^	450.20 ± 27.29^a^	2.84 ± 0.11^c^
M-LCQK	362.28 ± 19.26^a^	455.81 ± 97.10^a^	1.64 ± 0.10^a^

*Means within a column followed by different letters are significantly different (*p* < 0.05).*

### Compositions of Douzhir and Ma tofu Microbiota

**FIGURE 3:**
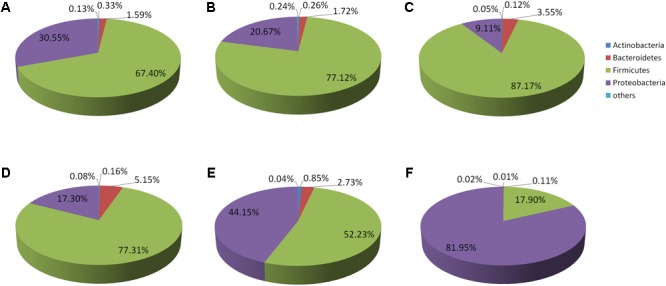
Distributions of the Douzhir and Ma tofu microbiota compositions at the phylum level. **(A)** D-DZ. **(B)** D-LFS. **(C)** D-LCQK. **(D)** M-DZ. **(E)** M-LFS. **(F)** M-LCQK. The respective proportions of each phylum in the D-DZ, D-LFS, and D-LCQK groups were as follows: Bacteroidetes: 1.59, 1.72, and 3.55%; Firmicutes: 67.4, 77.12, and 87.17%; Proteobacteria: 30.55, 20.67, and 9.11%; others: 0.13, 0.24, and 0.05%. The respective proportions of each phylum in the M-DZ, M-LFS, and M-LCQK groups were as follows: Bacteroidetes: 5.15, 2.73, and 0.11%; Firmicutes: 77.31, 52.23, and 17.9%; Proteobacteria: 17.3, 44.15, and 81.95%; others: 0.08, 0.04, and 0.02%.

Representatives of six phyla were found in 12 replicates of the six original samples. In the D-DZ samples, Firmicutes (67.4%) and Proteobacteria (30.55%) accounted for 97.95% of the total microbiota. In the D-LFS samples, Firmicutes (77.12%) and Proteobacteria (20.67%) comprised 97.79% of all bacteria. In the D-LCQK samples, Firmicutes (87.17%) and Proteobacteria (9.11%) accounted for 96.28% of the total microbiota. Thus, Douzhir samples from different manufacturers had similar microbiota, with Firmicutes being the most abundant phylum (over 67% of all species), followed by Proteobacteria and Bacteroidetes (**Figure 3**).

Firmicutes was also the most abundant phylum in the M-DZ and M-LFS samples, whereas in the M-LCQK samples, Proteobacteria representatives were most abundant (**Figure 3F**). In the M-DZ samples, Firmicutes (77.31%) and Proteobacteria (17.30%) accounted for 94.61% of all bacteria. In the M-LFS samples, Firmicutes (52.23%) and Proteobacteria (44.15%) represented 96.38% of all bacterial species. In the M-LCQK samples, Proteobacteria (81.95%) and Firmicutes (17.90%) comprised 99.85% of the total microbiota.

**FIGURE 4:**
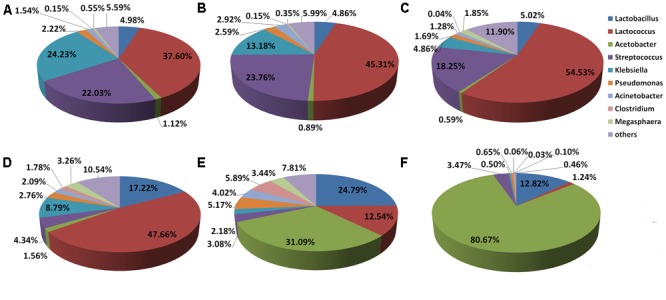
Distributions of the Douzhir and Ma tofu microbiota compositions at the genus level. **(A)** D-DZ. **(B)** D-LFS. **(C)** D-LCQK. **(D)** M-DZ. **(E)** M-LFS. **(F)** M-LCQK. The respective proportions of each genus in the D-DZ, D-LFS, and D-LCQK groups were as follows: *Lactobacillus*: 4.98, 4.86, and 5.02%; *Lactococcus*: 37.60, 45.31, and 54.53%; *Acetobacter*: 1.12, 0.89, and 0.59%; *Streptococcus*: 22.03, 23.76, and 18.25%; *Klebsiella*: 24.23, 13.18, and 4.86%; *Pseudomonas*: 2.22, 2.59, and 1.69%; *Acinetobacter*: 1.54, 2.92, and 1.28%; *Clostridium*: 0.15, 0.15, and 0.04%; *Megasphaera*: 0.55, 0.35, and 1.85%; others: 5.59, 5.99, and 11.90%. The respective proportions of each genus in the M-DZ, M-LFS, and M-LCQK group were as follows: *Lactobacillus*: 17.22, 24.79, and 12.82%; *Lactococcus*: 47.66, 12.54, and 1.24%; *Acetobacter*: 1.56, 31.09, and 80.67%; *Streptococcus*: 4.34, 3.08, and 3.47%; *Klebsiella*: 8.79, 2.18, and 0.50%; *Pseudomonas*: 2.76, 5.17, and 0.65%; *Acinetobacter*: 2.09, 4.02, and 0.06%; *Clostridium*: 1.78, 5.89, and 0.03%; *Megasphaera*: 3.26, 3.44, and 0.10%; others: 10.54, 7.81, and 0.46%.

**Figures 4A–C** shows the distribution of Douzhir microbiota at the genus level. In the D-DZ samples, the dominant genera included *Lactococcus, Klebsiella, Streptococcus*, and *Lactobacillus*, which accounted for 37.6, 24.23, 22.03, and 4.98% of the microbiota, respectively. The D-LFS samples were dominated by the same genera, but at slightly different proportions: *Lactococcus, Streptococcus, Klebsiella*, and *Lactobacillus* comprised 45.31, 23.76, 13.18, and 4.86% of the microbiota, respectively. In the D-LCQK samples, *Lactococcus, Streptococcus, Lactobacillus*, and *Klebsiella* were also the dominant genera, representing 54.53, 18.25, 5.02, and 4.86% of the microbiota, respectively. Thus, *Lactococcus, Streptococcus, Klebsiella*, and *Lactobacillus* were the predominant genera in Douzhir samples from all three examined manufacturers. *Lactococcus* was the most highly represented genus, as its species comprised between 37.6 and 54.53% of all bacterial species in the Douzhir samples.

The distribution of the Ma tofu microbiota at the genus level is illustrated in **Figures 4D–F**. In the M-DZ samples, the dominant genera included *Lactococcus, Lactobacillus*, and *Klebsiella*, which accounted for 47.66, 17.22, and 8.79% of the microbiota, respectively. The M-LFS samples were dominated by *Acetobacter, Lactobacillus*, and *Lactococcus*, which contributed 31.09, 24.79, and 12.54% to the total bacterial species, respectively. In the M-LCQK samples, *Acetobacter* and *Lactobacillus* species represented 80.67 and 12.82% of the microbiota, respectively. These data indicated that in the M-LFS and M-LCQK samples, *Acetobacter* was the most represented genus and its species comprised between 31.09 and 80.67% of total microbiota, respectively. In contrast, in the M-DZ samples, the most abundantly represented genus was *Lactococcus*. Thus, samples from all three Ma tofu manufacturers contained *Lactobacillus* species, and their contents were at least 10% of the total microbiota.

**FIGURE 5:**
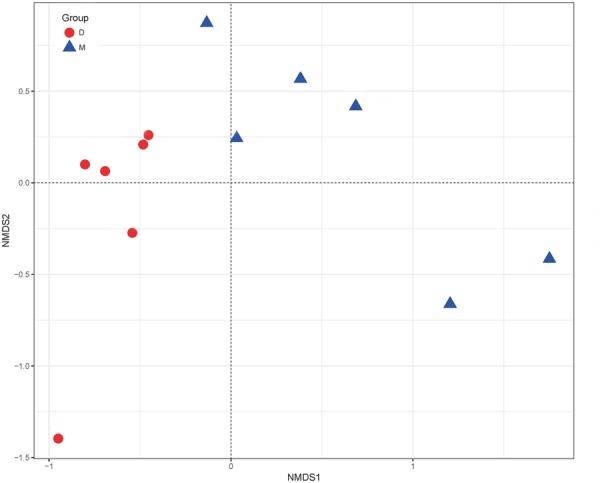
Non-metric multidimensional scaling (NMDS) ordination. NMDS plots demonstrating that Douzhir and Ma tofu harbored different bacterial communities.

The NMDS results showed differences in the microorganism distributions in the Douzhir and Ma tofu samples (**Figure 5**). The microorganisms in Douzhir clustered in one group, whereas those in Ma tofu clustered in another group.

### Isolation and Identification of Acidogenic, Starch-Flocculating Strains

Culturing of samples on mung bean juice-agar medium plates containing calcium carbonate led to the isolation of 56 strains that produced calcium carbonate clearance zones. Those strains were separated, added to liquid mung bean juice medium, and cultured for 24 h at 30°C. This experimental set up was established to assess the ability of these strains to coagulate starch and to identify strains that exhibited flocculating activity. By screening with fermentation liquid, we found that eight strains had high FR values and acidified the medium to pH 4.5 or lower (**Table 3**).

**Table 3:** Flocculation rate (FR) and pH measurements.

Strain	Individual form	Gram stain	FR (%) (*n* = 3)	pH (*n* = 3)
D-7	Bacillus	+	40.78 ± 0.41^a^	4.35 ± 0.02^d^
D-11	Cocci	+	49.72 ± 0.86^c^	4.36 ± 0.02^d^
D-23	Cocci	+	51.73 ± 0.41^d^	3.83 ± 0.03^b^
D-36	Bacillus	+	46.89 ± 0.32^b^	3.94 ± 0.02^c^
M-6	Bacillus	+	40.18 ± 0.27^a^	3.59 ± 0.05^a^
M-10	Bacillus	–	53.79 ± 0.43^e^	3.92 ± 0.03^c^
M-18	Bacillus	–	49.78 ± 0.83^c^	3.95 ± 0.03^c^
M-42	Cocci	+	41.26 ± 0.30^a^	4.31 ± 0.03^d^

*Means within a column followed by different letters are significantly different (*p* < 0.05).*

Eight strains were initially identified based on the features of their growth on mung bean juice agar medium, morphological characteristics, and gram staining. Six strains were gram positive, and two strains were gram negative. As shown in **Table 3**, strains D-23 and M-10 exhibited FRs higher than 50%, i.e., significantly higher than the FRs of other strains (*P* < 0.05). The pH of the fermentation liquid was below 4. Full-length 16S rRNA gene sequencing identified strain D-23 as *Lactococcus lactis* subspecies *lactis* (belonging to Firmicutes) and strain M-10 strain as *Acetobacter indonesiensis* (of the Proteobacteria phylum).

**FIGURE 6:**
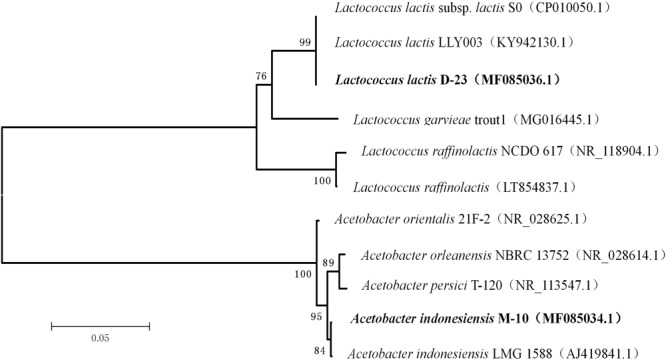
Phylogenetic relationships of D-23 and M-10 with related species based on partial 16S rDNA gene-sequence analysis. The phylogenetic tree was constructed using the neighbor-joining method (MEGA 5.0). The numbers at the nodes are bootstrap confidence levels (expressed as the percentage) from 1,000 replicates. The scale bar represents 0.05 substitutions per nucleotide position. Reference sequences were obtained from the GenBank nucleotide sequence database.

A phylogenetic tree was generated using the neighbor-joining method after aligning the nucleotide sequences of strains D-23 and M-10 (accession numbers MF085036 and MF085034.1, respectively) with sequences in the GenBank database (**Figure 6**). Strain D-23 formed a distinct cluster with *Lactococcus lactis*, as supported by a bootstrap value of 99%. Strain M-10 formed a distinct cluster with *Acetobacter indonesiensis*, as supported by a bootstrap value of 84%.

Strain D-23 was isolated from a Douzhir sample. As shown in **Figure 4**, in Douzhir samples from all three examined manufacturers, *Lactococcus* species were the dominant bacteria, and their content was the highest in all samples. Strain M-10 was isolated from a Ma tofu sample, and **Figure 4** shows that *Acetobacter* was the dominant genus in that sample. Thus, the D-23 and M-10 strains both originated from the dominant genera of the respective samples.

### Analysis of Starch Granules With Attached Bacteria

**FIGURE 7:**
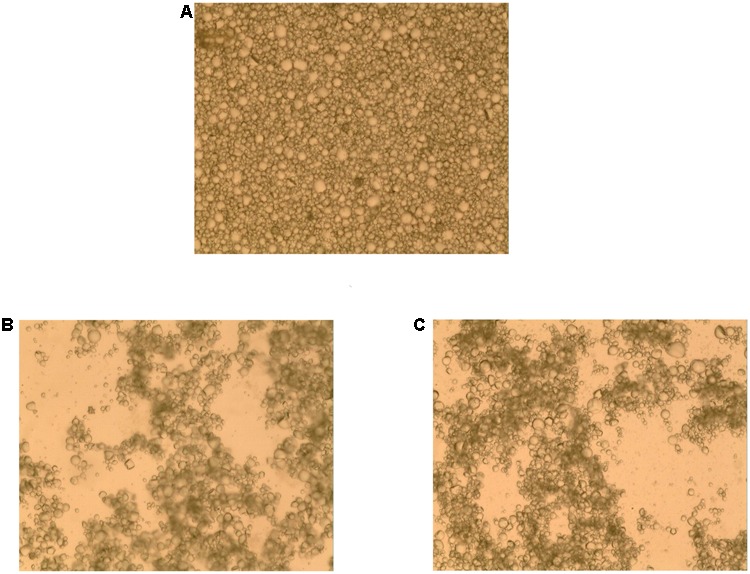
Optical micrograph of starch granule aggregation. **(A)** Un-inoculated mung bean starch milk. **(B)** Mung bean starch milk after the addition of D-23 strain fermentation liquid. **(C)** Mung bean starch milk after the addition of M-10 strain fermentation liquid.

**FIGURE 8:**
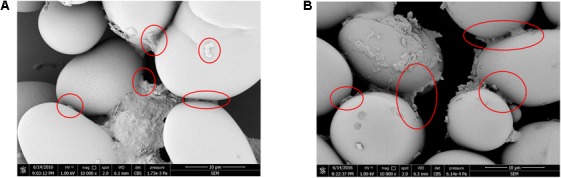
Scanning electron micrograph of starch granules with attached D-23 and M-10 cells. **(A)** D-23 strain (10,000×). **(B)** M-10 strain (10,000×).

To investigate bacterial adhesion to starch granules, the distribution of mung bean starch particles was observed before and after adding fermentation liquid from selected strains under an optical microscope. **Figure 7A** shows that starch granules were evenly distributed before the addition of fermentation liquid. **Figure 7B** shows the aggregation of starch granules into large flocs after adding the D-23 and M-10 fermentation liquids. Scanning electron microscopy revealed that bacterial cells bound the starch granule surface, and starch particles were bonded (**Figure 8**). Thus, starch granules coagulated into large flocs, and their enlargement caused accelerated starch precipitation due to gravity.

## Discussion

Because reliance upon spontaneous fermentation limits the standardization of industrial Douzhir production, selecting known fermentation strains is important (Park et al., 2011; Settanni et al., 2012). Acid formation and starch flocculation are two necessary conditions for Douzhir fermentation. To tackle these problems, high-throughput sequencing and traditional plate-based separation techniques were used to select acid-forming strains with starch-flocculating activity for their subsequent development as Douzhir-fermentation agents.

We performed high-throughput sequencing to determine the dominant microbiota in samples of naturally fermented Douzhir and Ma tofu from different manufacturers. We found that *Lactococcus, Streptococcus, Klebsiella*, and *Lactobacillus* were dominant genera that were responsible for most species identified in Douzhir. Representatives of the *Lactococcus* genus comprised the highest fraction of bacteria in all Douzhir samples. Among three Ma tofu samples, *Acetobacter* represented the dominant genus in two samples, and *Lactococcus* species dominated in one sample. Ma tofu samples from all three different sources contained *Lactobacillus*, whose content accounted for over 10% of all species. Although *Acetobacter* was dominant in the M-LFS and M-LCQK samples, it was rarely observed in Douzhir samples. Proteins are primary components of Ma tofu. After the addition of raw Douzhir to mung bean starch milk, the pH of the medium decreased and some proteins settled between the starch and bean juice. Bean juice mainly comprises soluble substances, so differences in the nutrient contents likely underpin distinct microbiota compositions of Douzhir and Ma tofu (Miao et al., 2013).

Dominant members of the Douzhir microbiota community have been analyzed previously by a traditional plate-culture technique, and 36 strains of lactic acid bacteria were isolated (Chen et al., 2013). In that study, it was concluded that Douzhir was mainly produced by lactic acid bacteria during lactic acid fermentation. *Lactococcus lactis* and *Lactobacillus curvatus* are major lactic acid bacteria. Ding et al. studied natural fermentation of mung bean juice under laboratory conditions and hypothesized that the natural acidification of bean juice primarily stems from acid production by bacteria (Ding et al., 2010). *Lactococcus lactis* and *Leuconostoc citreum* are major acid-producing microorganisms. Analysis of the microbiota of Douzhir by high-throughput sequencing and a traditional plate-culture method generated somewhat discrepant results. *Klebsiella, Streptococcus*, and *Acetobacter* were not detected by the plate-culture technique. This method is limited by the experimental conditions used, such as the selected medium and culture temperature, which may not be suitable for growth of all microorganisms. Because Douzhir is a product of natural fermentation, its microbiota is influenced by natural and operating conditions. Thus, data from this study and previous reports revealed that the bacterial species composition of Douzhir varied between different batches or manufacturers in different seasons (Riesenfeld et al., 2004; Hamady and Knight, 2009; Escobar-Zepeda et al., 2016).

In this study, the dominant microbiota in Douzhir and Ma tofu was represented by *Lactococcus, Acetobacter, Lactobacillus*, and *Streptococcus* species. The dominant bacteria were isolated and purified using mung bean juice as the growth medium. Using acid formation and the FR as criteria, two acid-forming bacteria (D-23 and M-10) were selected that met the fermentation requirements for Douzhir. After adding the fermentation liquids of these two strains, the starch granules rapidly aggregated and formed massive floccules (**Figure 7**). Furthermore, scanning electron microscopy analysis revealed that many bacterial cells adhered to the surface of starch granules and also to each other, forming bridge-like structures that interlinked starch granules and promoted floc aggregation (**Figure 8**).

*Lactococcus lactis* subsp. *lactis* has been previously isolated from sour liquid during processing of mung bean starch. Moreover, it was proposed that bacterial cells (rather than produced metabolites) accelerated mung bean starch sedimentation (Liu and Shen, 2007a,b). To the best of our knowledge, *Acetobacter indonesiensis* isolated in this study is the first acidogenic bacterium that promoted starch flocculation.

Starch-binding activity of microorganisms was also observed in other studies (Rodriguez-Sanoja et al., 2005; Olanipekun et al., 2009; Niderman-Meyer et al., 2010; Anderson et al., 2011; Nazarian-Firouzabadi et al., 2012). In 2006, Crittenden et al. discovered the starch-binding property of some *Bifidobacteria* species and *Lactobacillus amyloliquefaciens*. Niderman-Meyer et al. (2010) observed good efficacies of resistant starch-based drugs in treating acute gastroenteritis caused by *Vibrio cholerae* and discovered specific binding of that bacterium to the surface of resistant starch granules. That property was associated with faster elimination of *Vibrio cholerae* from the body. It was recently reported that *Bifidobacterium* species have strong starch-binding ability that allows them to become absorbed and embedded into resistant starch granules. Utilization of this binding in manufacturing resulted in a significant facilitation of probiotic microcapsule production (Crittenden et al., 2001).

Acid-forming strains with starch-binding activity identified in this study can be developed into Douzhir fermentation agents. In addition, the demonstrated starch-binding properties of these strains may be applied for improving technologies required for probiotic microcapsule production.

## Conclusion

Investigating the microbiota of Beijing Douzhir and Ma tofu showed that the dominant bacteria found in these food products belonged to Firmicutes and Proteobacteria, as these microorganisms comprised over 94% of the microorganisms in all samples. At the genus level, representatives of *Lactococcus, Acetobacter, Streptococcus*, and *Lactobacillus* comprised most of the identified genera. Two strains of acid-producing bacteria with starch-flocculating activity, *Acetobacter indonesiensis* and *Lactococcus lactis* subsp. *lactis*, were isolated from Douzhir and Ma tofu samples using a plate-separation technique. Such strains can serve as a foundation for standardized industrial production of Douzhir.

## Availability of Data and Material

The sequences obtained in this project were deposited in the NCBI sequence read archive (http://www.ncbi.nlm.nih.gov/traces/sra/) under SRA accession number SRP126032. Two strains of acid-producing bacteria with starch-flocculating activity were identified as *Acetobacter indonesiensis* and *Lactococcus lactis* subsp. *lactis* by 16S rDNA sequence homology comparisons. Sequences of 16S rDNA were deposited in the NCBI sequence read archive (https://www.ncbi.nlm.nih.gov/genbank/) under GenBank accession numbers MF085036 (D-23) and MF085034.1 (M-10).

## Author Contributions

LZ, YX, YY, and XL performed all the experiments and wrote the paper. YT, LL, JH, YS, HYZ, HJZ, and ZZ conducted the experiments and data analysis. All authors read and approved the manuscript.

## Conflict of Interest Statement

The authors declare that the research was conducted in the absence of any commercial or financial relationships that could be construed as a potential conflict of interest.

## Footnotes

1 http://www.tiangen.com/asset/imsupload/up0921879001368428871.pdf

2 http://ccb.jhu.edu/software/FLASH/

3 http://www.mothur.org/

4 http://qiime.org/scripts/pick_otus.html

5 http://greengenes.secondgenome.com/

6 www.ncbi.nlm.nih.gov/blast
